# Tofacitinib reduces acute lung injury and improves survival in a rat model of sepsis by inhibiting the JAK-STAT/NF-κB pathway

**DOI:** 10.1186/s12950-023-00332-3

**Published:** 2023-02-03

**Authors:** Xinxin Zhang, Xingsheng Wang, Li Sun, Guangsheng Gao, Yun Li

**Affiliations:** 1 Department of Emergency Medicine, Fuyang People’s Hospital of Anhui Medical University, Fuyang, Anhui China; 2 Intensive Care Unit, Central Hospital Affiliated to Shandong First Medical University, Jinan, Shandong China; 3 Department of Health Care, Central Hospital Affiliated to Shandong First Medical University, Jinan, Shandong China

**Keywords:** Sepsis, Cecum ligation perforation, Tofacitinib, JAK-STAT, NF-κ B

## Abstract

Acute lung injury is a major cause of death in sepsis. Tofacitinib (TOFA), a JAK inhibitor, has anti-inflammatory activity in autoimmune diseases, but its role in acute lung injury in sepsis remains unclear. The purpose of this study is to establish a septic rat model by cecal ligation and perforation, and to evaluate the effect of tofacitinib on the survival rate of septic rat model and its role in acute lung injury in septic rats and the possible mechanism of action. In this study, TOFA (1 mg/kg, 3 mg/kg, 10 mg/kg) was used to observe the survival rate of septic rats. It was found that TOFA (10 mg/kg) significantly improved the survival rate of septic rats. We selected TOFA (10 mg/kg) and focused on the protective effect of TOFA on acute lung injury. The results confirmed that TOFA significantly inhibited the expression of TNF-α, IL-1β, IL-6 and IFN-γ inflammatory factors, reduced the W/D weight ratio of septic lung tissue, and significantly improved lung histopathological damage. These results may be related to the inhibitory effect of TOFA on JAK-STAT/NF-κ B signaling pathway. In conclusion, for the first time, we found that TOFA has a protective effect against sepsis-induced acute lung injury, and it may be a promising drug for the treatment of acute lung injury in sepsis.

## Introduction

Sepsis is defined as a life-threatening organ dysfunction due to a dysregulated host response to infection [23, 28]. The incidence of sepsis is high, with more than 18 million cases of severe sepsis worldwide each year, and increasing at a rate of 1.5% to 8.0% per year. Sepsis is a fatal disease, with approximately 14,000 deaths per day worldwide due to sepsis complications [16]. Sepsis currently affects one-fifth of all ICU patients in China, with a 90-day mortality rate of 35.5% [33]. Compared to non-sepsis, sepsis is associated with a higher mortality rate as well as higher health care costs, even with higher readmission rates after discharge than non-septic diseases [11]. The high prevalence and mortality of sepsis remains a huge problem for humanity, so further research into the pathophysiological mechanisms of sepsis and the search for new treatment modalities are urgent scientific research questions.

The development of sepsis occurs in two stages:① Systemic (high) inflammatory response syndrome (SIRS), which is involved in multi-organ failure and is the cause of death in the developmental stage of sepsis [3, 10].② Compensatory anti-inflammatory response syndrome (CARS), which leads to sepsis-induced immunosuppression and is the main cause of secondary infections and late mortality [1, 30]. The JAK-STAT signaling pathway is involved in both phases and plays a key role in sepsis [7]. The current study confirmed the involvement of the JAK-STAT pathway in cecum ligation perforation (CLP)-induced multiorgan damage in septic rats [9]. During sepsis, lipopolysaccharide upregulates JAK-STAT/NF-κ B pathway expression and increases the release of inflammatory mediators (IL-6, TNF-α and IL-10) through Toll-like receptor interactions with innate immune cells during sepsis, leading to alveolar epithelial cell and vascular endothelial cell injury, which in turn causes diffuse acute lung injury [4, 25]. The JAK-STAT pathway is also an effective therapeutic target for various cytokine-driven autoimmune and inflammatory diseases [19]. Therefore, inhibition of the JAK-STAT pathway becomes a potential therapeutic route for sepsis.

Tofacitinib (TOFA) is a JAK inhibitor that blocks the signal transduction of various inflammatory cytokines by blocking the JAK-STAT signaling pathway, and has been approved by the Food and Drug Administration (FDA) for the treatment of rheumatoid arthritis since 2012. Previous studies have also proved that TOFA has good therapeutic effects on a variety of inflammation-related diseases such as ulcerative colitis, psoriasis [12] and pemphigus vulgaris [14] by inhibiting the JAK-STAT pathway [26]. However, its role in acute lung injury in sepsis remains unclear.

The aim of this study was to elucidate the effect of TOFA on survival in a rat model of sepsis, the role and possible mechanism of TOFA in acute lung injury of sepsis rats, so as to provide a theoretical basis for the use of TOFA in the treatment of sepsis.

## Materials and methods

### Experimental animals

Male SD rats were 8–10 weeks old and weighed 250-300 g, and were acclimatized and housed for one week. The experimental protocol was approved by the Experimental Animal Management and Ethics Committee of the Central Hospital Affiliated Shandong First Medical University.

### CLP model preparation

The operation procedure was mainly based on the study of Rittirsch [24]. The animal model of sepsis was established using cecum ligation perforation (CLP). 1% sodium pentobarbital (50 mg/kg, i.p.) was used for anesthesia, and the cecum was exposed by a midline abdominal incision. The cecum was separated from the cecum branch of the ileocecal artery and ligated with a 4–0 silk thread 3/4 of the way from the end of the cecum. An 18-gauge needle was used twice through the cecum to squeeze a tiny amount of feces from each opening. The sham operation group underwent the same operation except ligation and cecal puncture.

### Experimental groups and intervention

Survival experiments were first performed, and 50 rats were randomly divided into 5 groups (*n* = 10): sham operation (Sham) group, CLP group, CLP + TOFA (1 mg/kg) group, CLP + TOFA (3 mg/kg) group, and CLP + TOFA (10 mg/kg) group. Two hours after modeling, TOFA (1, 3 or 10 mg/kg) or solvent was given intragastric administration every 6 h. The survival status and survival rate of the rats were monitored daily for 7 days. In the mechanistic exploration, the dose (10 mg/kg) that had a better effect on survival was selected. Thirty rats were randomly divided into three groups, sham operation (Sham) group, CLP group, and CLP + TOFA (10 mg/kg) group. 24 h after model establishment, blood and lung tissue samples were collected from anesthetized rats. TOFA was prepared under sterile conditions at the ratio of 5% DMSO, 30% PEG-300, 5% Tween 80, and 60% saline before use.

### Lung histological analysis

Twenty four hours after model establishment, rats were anesthetized, and their unilateral lung tissues were fixed in 4% paraformaldehyde solution for 24 h. After dehydration, they were embedded in paraffin, sliced into 5 μm sections, and the pathological changes were observed by hematoxylin–eosin (H&E) staining. Lung tissue damage was scored by a professional pathologist under double-blind conditions based on the structural integrity of the alveolar cavity, neutrophil and lymphocyte infiltration, interstitial edema of the alveolar cavity, and hyaluronic membrane formation. The scoring criteria were: no lung tissue damage, 0 points; lung tissue damage range < 25%, 1 point; lung tissue damage range 20–50%, 2 points; lung tissue damage range 50–75%, 3 points; lung tissue damage range > 75%, 4 points. Four different views of each pathological section were selected for scoring, and the results were statistically analyzed.

### Lung wet/dry (W/D) weight ratio

The right lung tissue of rats was taken with absorbent paper to remove the surface water, weighed and recorded as wet weight. The tissue was then baked in a drying oven at 60℃ for 72 h until the weight remained constant, weighed and recorded as dry weight. The W/D weight ratios were calculated.

### ELISA

Total protein was extracted from lung tissue homogenates and assayed for IL-6, TNF-α, IL-1β, IFN-γ using commercially available ELISA kits (Boster Biotechnology, USA). Absorbance was measured at 450 nm using a VersaMax enzyme marker. The concentrations were calculated by using a standard curve generated from standard proteins.

### RT-qPCR

The expression levels of IL-6, TNFα, IL-1β and IFN-γ were measured using RT-qPCR. Total RNA was extracted from homogenized lung tissue using the Trizol kit (BioTeke, CHN) according to the instructions. Reverse transcription of each group of RNA with reverse transcription kit (BioTeke, CHN) to obtain corresponding cDNA. SYBRGreen reagent was used to analyze gene expression in Lightcycler96PCR system. GAPDH was used as internal control. Each sample is tested at least three times and averaged. The primer sequences used for RT-qPCR are shown in Table 1.

**Table 1:** Primers of RT-qPCR analysis (5’ → 3’)

Gene	Forward primer	Reverse primer
IL-6	5’-ATGGCAATTCTGATTGTATG-3’	5ʼ-GACTCTGGCTTTGTCTTTCT-3’
TNF-α	5’-CCAGACCCTCACACTCACAAA-3’	5ʼ-GGCTGACGGTGTGGGTGAG-3’
IL-1β	5’-GGGATGATGACGACGACCTGCTA-3’	5ʼ-ACAGCACGAGGCATTTTTGT-3’
IFN-γ	5’-GAGGAACTGGCAAAAGGACG-3’	5ʼ-AGGTGCGATTCGATGACACT-3’
Gapdh	5’-GAGGAACTGGCAAAAGGACG-3’	5’-AGGTGCGATTCGATGACACT-3’

### Western blotting

Lung tissue was lysed in RIPA lysate and phosphatase inhibitor, ground on ice and centrifuged at 4 °C for 10 min at 12,000 rpm, and protein concentration was determined using a BCA kit (Beyotime, CHN). Samples were denatured at 100 °C for 10 min and stored at -80 °C overnight. The proteins were separated on the gel by electrophoresis and transferred to PVDF membranes, which were then closed with 5% BSA TBST at room temperature for 1 h. The proteins were then washed 3 times effectively with TBST and incubated with diluted primary antibody overnight at 4 °C. The antibody was again washed 3 times with TBST and incubated with secondary antibody at room temperature for 1 h. The protein band was developed with extremely sensitive chemiluminescence reagent and finally analyzed for grayscale values using ImageJ software. Anti-STAT3 (4904 T, 1:2000, CST, USA), anti-p-STAT3 (9145 T, 1: 2000, CST, USA), anti-NF-κ B (8242, 1: 1000, CST, USA), anti-p-NF-κ B (3033, 1: 1000, CST, USA), and anti-GAPDH (5174, 1:1000, CST, USA) antibodies were obtained from CST.

### Statistical analysis

Data analysis and drawing are carried out using SPSS 26.0 and GraphPadPrism 8.0 software. The quantitative data were expressed by mean ± standard deviation, the data of multi-group samples were compared by single-factor analysis of variance (ANOVA), and the data of the two groups were compared by double-tailed unpaired *t* test.

## Results

### TOFA improves survival and viability in septic rats

**Fig. 1:**
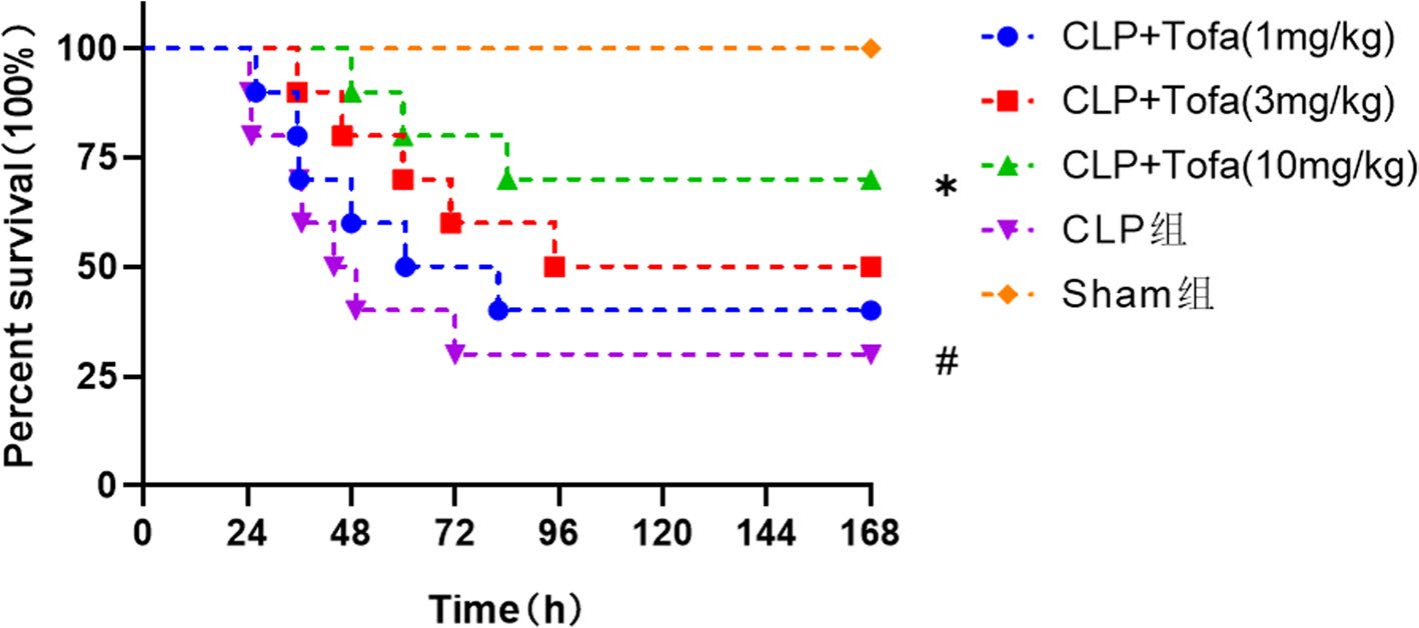
Results of survival analysis in 5 groups of rats. The experiment was divided into 5 groups: Sham group, CLP group, CLP + Tofa (1 mg/kg) group, CLP + Tofa (3 mg/kg) group, CLP + Tofa (10 mg/kg) group, After surgery, the rats were continuously observed for 7 days, and their status and survival were recorded, and the survival rates of each group were compared. (*P < 0.05 *vs*. CLP group, #*P* < 0.05 *vs*. Sham group)

Monitoring the survival of each group of rats 7 days after surgery. The survival rate was 100% in the Sham group and only 30% in the CLP group compared with the Sham group (*P* < 0.05). The survival rate of CLP + TOFA (1 mg/kg) group was 40%, and that of CLP + TOFA (3 mg/kg) group was 50%, which had no statistical difference compared with CLP group (*P* > 0.05). The survival rate in the CLP + TOFA (10 mg/kg) group was 70%, with a significantly higher survival rate compared to the CLP group (*P* < 0.05) (Fig. 1). After CLP surgery or sham surgery, the rats in the Sham group returned to their normal state soon after surgery in terms of food and water intake, activity level, and mental status. The CLP group showed decreased water intake, decreased activity, oozing of blood from the eyes, poor mental status, unresponsiveness, inverted dorsal hair and loss of luster, and decreased urination and defecation, while the rats in the CLP + TOFA (10 mg/kg) group showed increased water intake, increased activity, active mental and no ocular oozing status compared with the CLP group. These results confirmed the protective effect of TOFA (10 mg/kg) on septic rats. Based on these results, we used TOFA (10 mg/kg) in the subsequent experiments.

### TOFA treatment reduces lung tissue injury

**Fig. 2:**
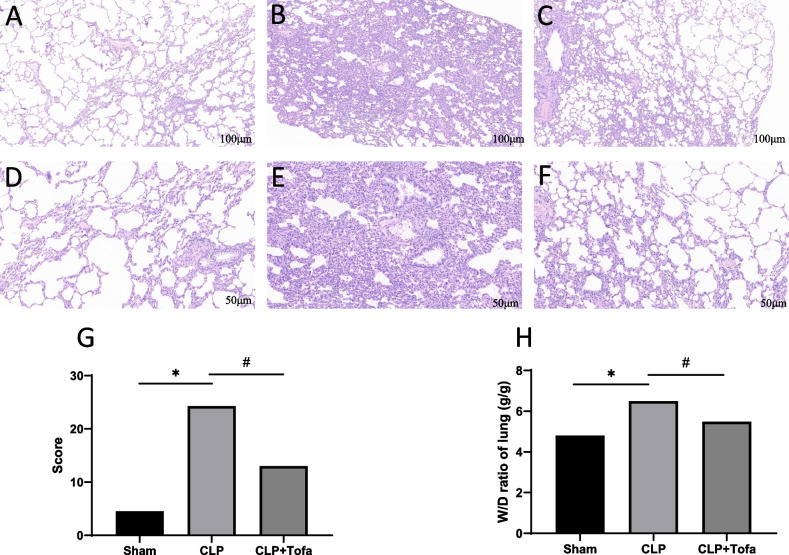
Histopathological changes, pathological damage scores, and W/D weight ratio of lungs in SD rats in each group after CLP. The experiment was divided into three groups Sham group, CLP group, and CLP + TOFA group. The lung tissues of SD rats were taken for **HE** staining after model establishment 24 h, and the pathological damage and W/D weight ratio were evaluated. **ABC** and **DEF** were lung tissue HE staining sections of Sham group, CLP group and CLP + TOFA group at 100 × and 200 × respectively. **G** is the lung damage score of rats. **H** is the W/D weight ratio of lungs. (*P < 0.05 *vs*. Sham group, #*P* < 0.05 *vs*. CLP group)

Microscopically, compared with the Sham group, the CLP group showed disorganized alveolar structure, a large number of neutrophil and lymphocyte infiltration in the alveolar cavity and septum, widening of the alveolar septum, and collapse of the alveolar cavity by compression. Similar pathological changes were observed in the lung tissue of the CLP + TOFA (10 mg/kg) group as in the CLP group, but the severity was relatively less (Fig. 2 A-F). The pathological scores of lung histomorphology in each group were subsequently performed. The pathological scores in the CLP group were significantly higher than those in the Sham group (*P* < 0.05). And the pathological score in the CLP + TOFA (10 mg/kg) group was significantly lower than that in the CLP group (*P* < 0.05) (Fig. 2 G). The results of lung W/D weight ratio showed that the W/D weight ratio was significantly increased in the CLP group compared with the Sham group (*P* < 0.05), and significantly decreased in the CLP + TOFA (10 mg/kg) group compared with the CLP group (*P* < 0.05) (Fig. 2 H). The results suggest that TOFA reduces the acute lung injury in CLP septic rats.

### TOFA reduces the expression of pro-inflammatory cytokines in lung tissue

**Fig. 3:**
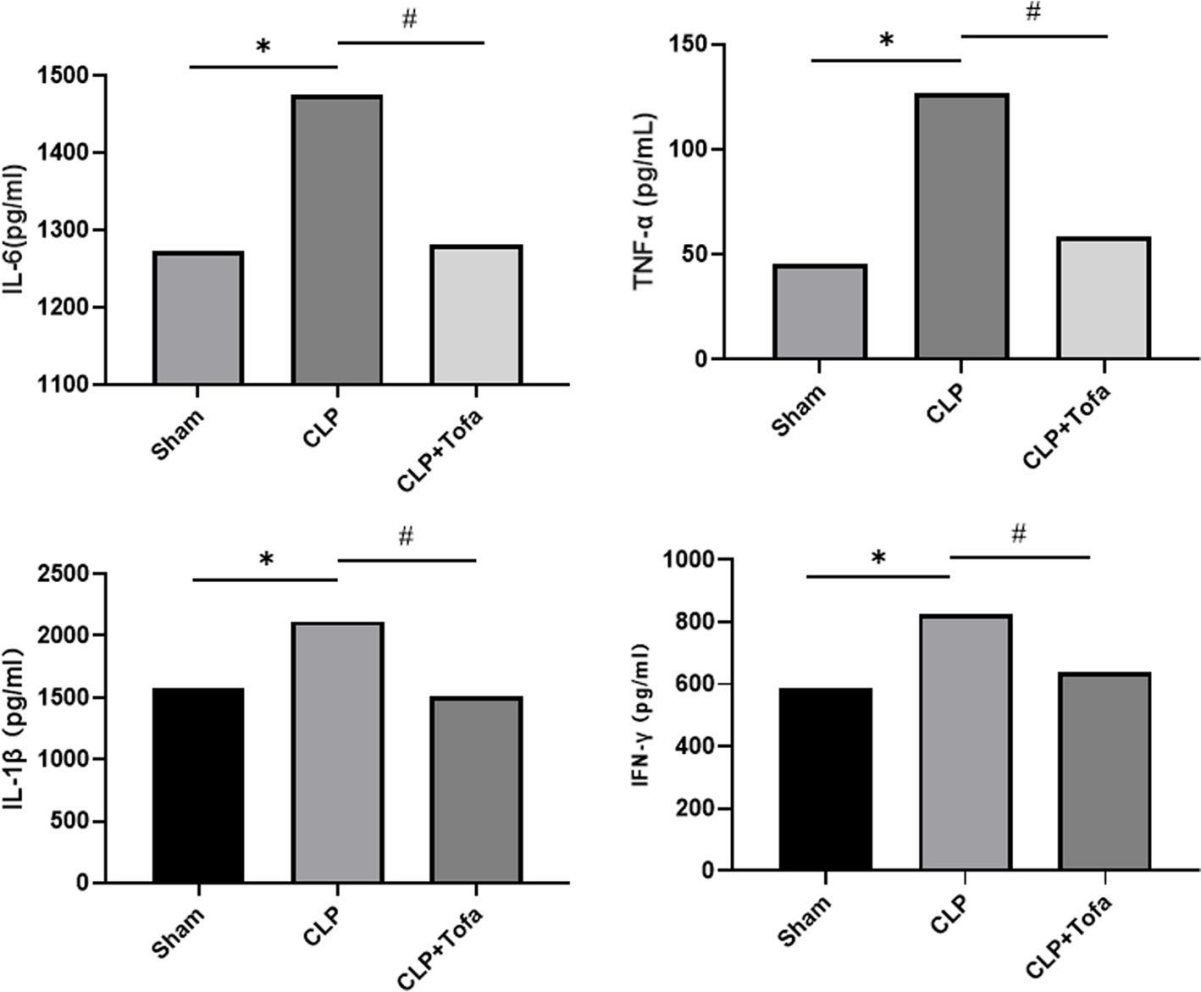
Expression of inflammatory factors in lung tissues of SD rats in each group was determined. ELISA was performed to detect the expression levels of IL-6, TNF-α, IL-1β and IFN-γ in rat lung tissues. (*P < 0.05 *vs*. Sham group, #*P* < 0.05 *vs*. CLP group)

**Fig. 4:**
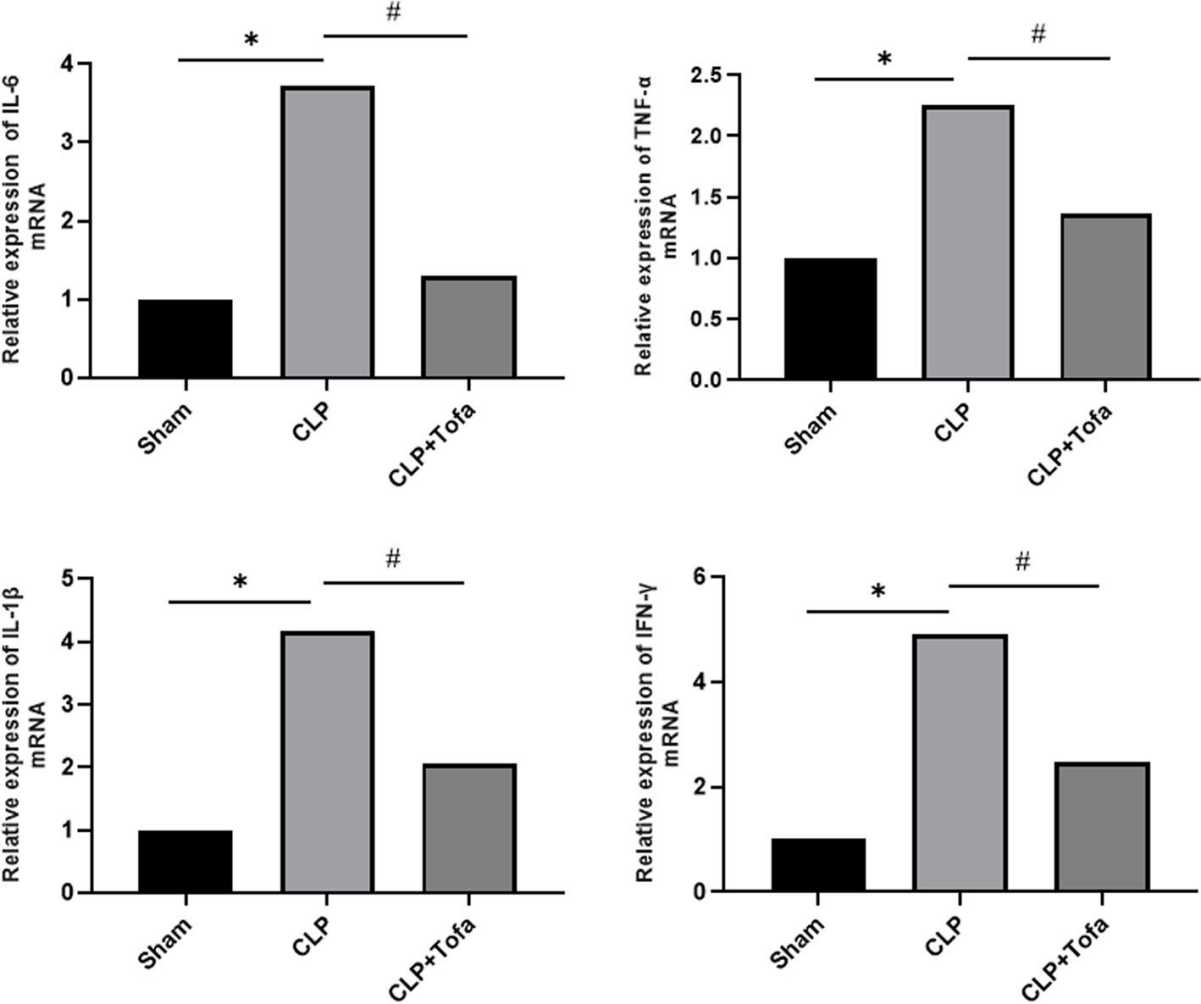
Expression of inflammatory factors in lung tissues of SD rats in each group was determined. RT-qPCR was performed to detect the expression levels of IL-6, TNF-α, IL-1β and IFN-γ in rat lung tissues. (*P < 0.05 *vs*. Sham group, #*P* < 0.05 *vs*. CLP group)

To determine the expression of inflammatory factors in rats, we used ELISA and RT-qPCR to detect the levels of pro-inflammatory cytokines in lung tissue. As shown in Fig. 3, IL-6, TNF-α, IL-1β and IFN-γ inflammatory factor expression was significantly increased in the CLP group compared with Sham (*P* < 0.05). Increased IL-6, TNF-α, IL-1β and IFN-γ expression was significantly inhibited in the CLP + TOFA (10 mg/kg) group (*P* < 0.05). As shown in Fig. 4, the results of IL-6, TNF-α, IL-1β and IFN-γ expression in lung tissues detected by RT-qPCR were consistent with ELISA, which indicated that TOFA inhibited CLP-induced production of pro-inflammatory cytokines in lung tissues (*P* < 0.05).

### TOFA treatment inhibited the activation of JAK-STAT3 and NF-κB signaling pathways

**Fig. 5:**
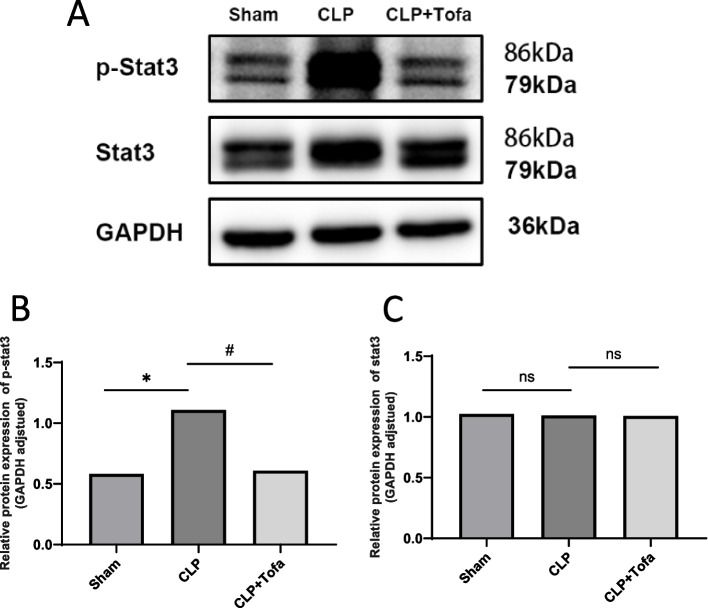
Detection of STAT3 and p-STAT3 expression levels in rat lung tissues. **A** Protein bands in 3 groups of rats; **B** Expression measurement of p-STAT3 protein in 3 groups of rats; **C** Expression measurement of STAT3 protein in 3 groups of rats. (*P < 0.05 *vs*. Sham group, #*P* < 0.05 *vs*. CLP group, ns: indicates no statistical difference)

**Fig. 6:**
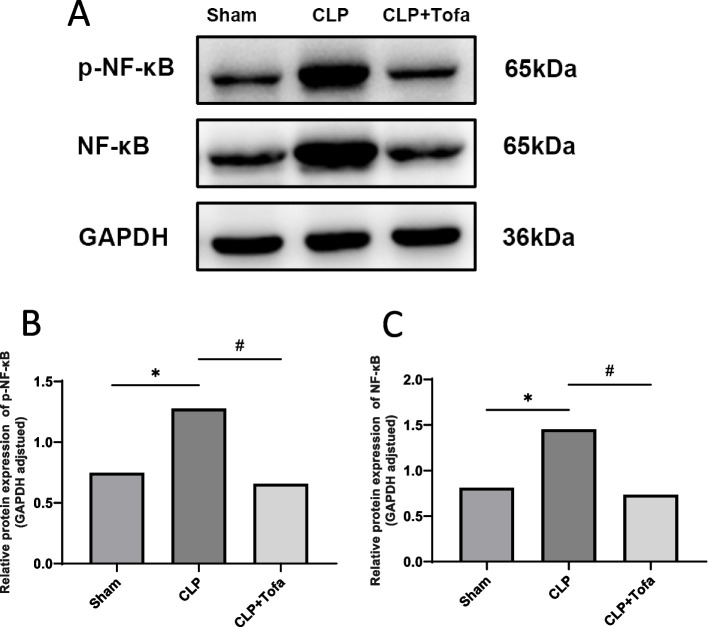
Detection of NF-κ B and p-NF-κ B expression levels in rat lung tissues. **A** Protein bands in 3 groups of rats; **B** Expression measurement of p-NF-κ B protein in 3 groups of rats; **C** Expression measurement of NF-κ B protein in 3 groups of rats. (*P < 0.05 *vs*. Sham group, #*P* < 0.05 *vs*. CLP group)

Western blot was used to study the effect of TOFA on JAK-STAT signaling pathway in CLP rats. As shown in Fig. 5, the percentage of STAT3 phosphorylation was significantly higher in the CLP group compared with the Sham group (*P* < 0.05). TOFA (10 mg/kg) group decreased the CLP-induced increase in STAT3 phosphorylation. We also study the effect of TOFA on NF-κ B signaling pathway. The results are shown in Fig. 6, p-NF-κ B levels were significantly increased in the CLP group compared with the Sham group (*P* < 0.05), and p-NF-κ B expression was significantly decreased in the TOFA (10 mg/kg) group compared with the CLP group. Both signaling pathways had similar trends.

## Discussion

Sepsis leading to organ dysfunction, especially acute lung injury, is very common in the clinic. Despite advances in strategies to manage and treat sepsis, morbidity and mortality in clinical care continue to climb [20]. We studied the survival rate of septic rats using TOFA (1 mg/kg, 3 mg/kg, 10 mg/kg) and found that TOFA (10 mg/kg) significantly increased the survival rate of septic rats. We chose TOFA (10 mg/kg) and focused on the protective effect of TOFA on acute lung injury. The results showed that TOFA significantly reduced the severity of lung histopathological injury and pulmonary edema in septic rats, and TOFA significantly down-regulated the expression of inflammatory factors and activation of downstream signaling pathways in septic rats.

Three doses of TOFA (1 mg/kg), TOFA (3 mg/kg) and TOFA (10 mg/kg) was used to intervene in septic rats. And the results showed that TOFA (10 mg/kg) improved the survival status of CLP rats and significantly increased the survival rate. The TOFA (1 mg/kg) and TOFA (3 mg/kg) also improved the survival rate of septic rats compared with the CLP group, but there was no statistical difference, and the reason was considered to be related to the small sample size.

In this experiment, we found that the expression of pro-inflammatory cytokines (TNF-α, IL-1β, IL-6 and IFN-γ) was significantly increased in the lung tissue of septic rats. Acute lung injury can be divided into exudative stage and fibroproliferative stage. The accumulation of neutrophils in the lung with a large number of pro-inflammatory cytokines during exudative stage is the main cause of acute lung injury, which can lead to the destruction of alveolar epithelial and endothelial barriers and induce the release of other inflammatory mediators. The hyperplastic stage is characterized by disordered healing and hyperplasia of fibrous tissue [29]. Numerous studies have also shown that excessive release of pro-inflammatory cytokines, such as IL-1β, IL-6 and TNF-α, triggers pathophysiological abnormalities in sepsis and plays an important role in septic lung injury [18, 22]. By using both ELISA and qPCR, we confirmed that the expression of TNF-α, IL-1β, IL-6 and IFN-γ inflammatory factors was significantly inhibited after TOFA intervention in septic rats. It was also found that TOFA reduced the CLP-induced W/D weight ratio of lung tissue. This finding was supported by histological analysis of lung tissue from CLP-induced sepsis rats, which showed a large infiltration of neutrophils and lymphocytes in the alveolar cavity and alveolar septum, thickness of the alveolar septum, disorganization of alveolar structures, and collapse of the alveolar cavity into a smaller size by compression. These findings suggest that TOFA may prevent the development of pulmonary edema and the infiltration of inflammatory cells into the lung, which is an important feature in the pathogenesis of acute lung injury [21].

**Fig. 7:**
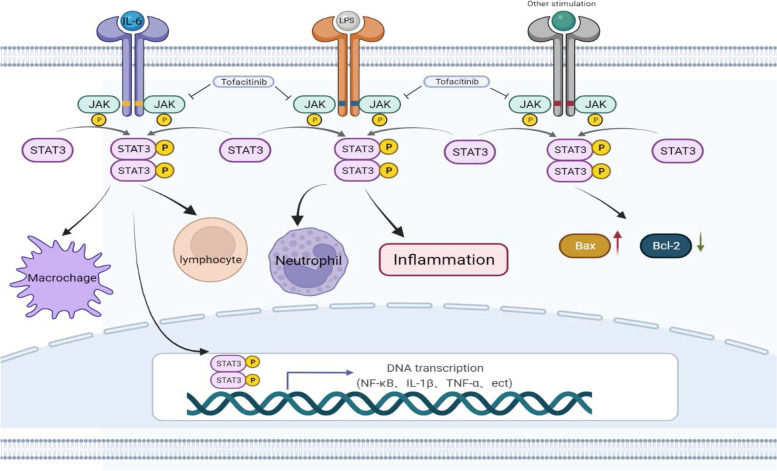
Activation of the JAK-STAT3 pathway. When inflammatory factors bind to receptors on the cell membrane, they induce receptors dimer formation and phosphorylation of JAK. Activated JAK can activate STAT3 in the cytoplasm to phosphorylate it, and the phosphorylated STAT3 binds to each other through the SH2 structural domain to form a dimer, which then enters the nucleus to bind to promoters to regulate gene expression

The JAK-STAT pathway is a major signaling pathway for many key cytokines signaling in sepsis. When JAK-STAT is activated by different sources of inflammatory factors, the inflammatory factors induce receptors dimer formation and phosphorylation of JAK when they bind to receptors on the cell membrane, and the activated JAK is able to activate STAT3 in the cytoplasm to phosphorylate it, and the phosphorylated STAT3 binds to each other through the SH2 structural domain to form a dimer, which then enters the nucleus or mitochondria to regulate target gene expression [8, 17] (Fig. 7). It has been shown that the STAT3 pathway is closely associated with inflammatory diseases including sepsis [6], and inhibition of the JAK-STAT3 signaling pathway is expected to improve the prognosis of sepsis. Previous studies have demonstrated that miR-210 intervention attenuates CLP-induced renal injury, inflammatory response and apoptosis in septic rats by inhibiting the JAK-STAT pathway [34, 35]. Emodin activates the JAK-STAT3 pathway, which increases p-STAT3 levels and protects the jejunum in septic rats by inhibiting inflammation [5]. In addition, TOFA treatment was found to aggravate staphylococcus aureus infectious arthritis but reduce sepsis and enterotoxin-induced shock in mice [15]. TOFA ameliorates lipopolysaccharide-induced acute kidney injury by blocking the JAK-STAT1/STAT3 signaling pathway [34]. However, studies related to TOFA treatment of lung injury in septic rats were not identified. Our results revealed that the JAK-STAT pathway was activated in septic rat lung tissue, with a significant increase in p-STAT3 expression, and that TOFA treatment significantly inhibited STAT3 phosphorylation. Other recent studies have demonstrated that inhibition of the STAT3 pathway protects the lung from injury (Jian [31]. The inhibition of JAK-STAT3 pathway by ulinastatin is related to the decrease of inflammatory mediators, thus reducing the necrosis and swelling of lung tissue in septic rats. We also found that TOFA also inhibited the activation of the NK-κ B pathway in lung tissue of septic rats. The expression of p-NF-κ B was significantly increased in the CLP group compared with the Sham group, whereas p-NF-κ B was significantly reduced after treatment with TOFA (10 mg/kg), indicating that inhibition of the JAK-STAT3 pathway was accompanied by inhibition of the NF-κ B pathway. It has been shown that JAK-STAT3 inhibitors can reduce the DNA binding activity of NF-κ B, suggesting that JAK-STAT3 signaling is upstream of the NF-κ B signaling pathway and that STAT3 can positively regulate the activation of NF-κ B [13]. This could be attributed to the regulation of NF-κ B signaling pathway by JAK-STAT3, or it could be a complex crosstalk between different signaling pathways.

However, it has also been reported that the activation of JAK-STAT3/NF-κ B pathway can enhance the host inflammatory response, and application of recombinant human interleukin-7 (RHIL-7) can increase the expression of STAT3/NF-κ B signaling pathway during the immunosuppression stage of sepsis, thus enhancing the clearance of pulmonary pathogens in CLP-induced sepsis [27].

According to the detection of sepsis biomarkers to determine the subtype of sepsis (immunosuppressive or high inflammatory response stage), the corresponding treatment at different stages may bring greater benefits to patients [29]. Previous studies have shown that inhibition of STAT3 phosphorylation levels during the hyperinflammatory phase of sepsis and increasing STAT3 phosphorylation levels during the immunosuppressive phase both improve pathogen clearance and improve healing in septic rats. This is consistent with the results of clinical studies that the use of hydrocortisone combined with fludrocortisone during the hyperinflammatory phase of sepsis reduces mortality in patients with sepsis at 90 days [2], and the use of thymidine α1 to enhance immunity during the immunosuppressive phase reduces mortality at 28 days [32].

In conclusion, TOFA can improve the survival rate of sepsis rats and improve the degree of acute lung injury in sepsis rats. Therefore, the application of TOFA in clinical sepsis patients at the stage of high inflammation of sepsis is expected to improve the prognosis of patients and improve the survival time of critically ill patients.

## Conclusion

In conclusion, the results of this study showed that TOFA (10 mg/kg) treatment significantly improved the survival status of CLP rats and increased the survival rate of rats. For the first time, TOFA was shown to play a protective role in the lung tissue of CLP-induced sepsis model by inhibiting the JAK-STAT/NF-κ B signaling pathway. Thus, TOFA is promising as a clinical candidate for the treatment of the hyperinflammatory phase of sepsis.

## Data Availability

All data are available for publication.
